# Isolation, Identification and Pigment Analysis of Novel Cyanobacterial Strains from Thermal Springs

**DOI:** 10.3390/plants13212951

**Published:** 2024-10-22

**Authors:** Sandugash K. Sandybayeva, Bekzhan D. Kossalbayev, Bolatkhan K. Zayadan, Jiří Kopecký, Ardak B. Kakimova, Kenzhegul Bolatkhan, Suleyman I. Allakhverdiev

**Affiliations:** 1 Faculty of Biology and Biotechnology, Al-Farabi Kazakh National University, Al-Farabi 71, Almaty 050038, Kazakhstan; sandybaevasandugash@gmail.com (S.K.S.); bkenzhegul23@gmail.com (K.B.); 2 Department of Chemical and Biochemical Engineering, Geology and Oil-Gas Business Institute Named after K. Turyssov, Satbayev University, Satpaev 22, Almaty 050043, Kazakhstan; kossalbayev.bekzhan@gmail.com; 3 Tianjin Institute of Industrial Biotechnology, Chinese Academy of Sciences, No. 32, West 7th Road, Tianjin Airport Economic Area, Tianjin 300308, China; 4 Ecology Research Institute, Khoja Akhmet Yassawi International Kazakh-Turkish University, Turkistan 161200, Kazakhstan; 5 Laboratory of Algal Biotechnology, Centre ALGATECH, Institute of Microbiology, Czech Academy of Sciences, Novohradská 237—Opatovický mlýn, 37981 Třebon, Czech Republic; kopecky@alga.cz; 6 K.A. Timiryazev Institute of Plant Physiology, Russian Academy of Sciences, Botanicheskaya Street 35, Moscow 127276, Russia; suleyman.allakhverdiev@gmail.com

**Keywords:** cyanobacteria, identification, extraction of pigments, biological activities, chromatography, carotenoids, myxoxanthophyll

## Abstract

Cyanobacterial pigments have attracted considerable attention in industry due to their bioactive potential and natural origin. In the present study, the growth dynamics and pigment composition, in terms of chlorophyll *a*, total carotenoids and phycobiliprotein content, of four cyanobacterial strains isolated from thermal springs, namely *Oscillatoria subbrevis* CZS 2201, *Phormidium ambiguum* CZS 2205, *Nostoc calcicola* TSZ 2203, and *Synechococcus* sp. CZS 2204, were investigated. The analysis revealed that the maximum quantity of chlorophyll *a* and total carotenoids was observed in *Oscillatoria subbrevis* CZS 2201 (26.49 and 3.44 µg mL^−1^), followed by *Phormidium ambiguum* CZS 2205 (18.64 and 2.32 µg mL^−1^), whereas a minimum amount was detected in *Synechococcus* sp. CZS 2204 (12.13 and 1.24 µg mL^−1^), respectively. In addition, *Oscillatoria subbrevis* CZS 2201 showed higher quantity of phycobiliproteins, especially C-phycocyanin (45.81 mg g^−1^), C-phycoerythrin (64.17 mg g^−1^) and C-allophycocyanin (27.45 mg g^−1^). Moreover, carotenoid derivatives of *Oscillatoria subbrevis* CZS 2201 were also identified, among which β-carotene was the dominant form (1.94 µg mL^−1^), while the accumulation of zeaxanthin and myxoxanthophyll was relatively high (0.53 and 0.41 µg mL^−1^, respectively) compared with echinenone and cryptoxanthin (0.34 and 0.23 µg mL^−1^, respectively). The study revealed that *Oscillatoria subbrevis* CZS 2201 was a potent producer of secondary carotenoids, including myxoxanthophyll.

## 1. Introduction

Ongoing concerns about food security, energy crisis, disease outbreaks, global warming and the need to reduce carbon dioxide emissions have challenged scientists to find environmentally friendly and renewable biological resources from living organisms for food, energy and materials [1]. There is a growing awareness among consumers to use natural products derived from microorganisms and plant-based materials to create new technologies for functional products that can contribute to the industrial bioeconomy [2]. Photosynthetic microorganisms, including cyanobacteria, are useful to produce highly valuable metabolites, including lipids, proteins, pigments and carbohydrates [3].

Cyanobacteria, which inhabit different environments and possess a wide range of metabolic capabilities, are characterized by their ability to produce a spectrum of biologically active compounds with antibacterial, antifungal, antiviral and antioxidant properties of pharmaceutical and agricultural importance [4,5]. Among these bioactive compounds, natural organic pigments such as chlorophylls, carotenoids (carotenes and xanthophylls) and phycobiliproteins (c-phycocyanin, allophycocyanin and c-phycoerythrin) are gaining importance in several cosmetic, food and textile industries [6,7]. Chlorophyll is the most fundamental; it is responsible for oxygenic photosynthetic activity and is present in all photosynthetic microalgae and cyanobacteria [8]. Carotenoids can be either primary or secondary pigments, and their profiles vary according to species and growth conditions; the best known and commercially available are astaxanthin, lutein and β-carotene [9]. In addition, phycobiliproteins are a special class of pigments found only in cyanobacteria and red algae and may be the most important light absorbers in these organisms; the best-known phycobiliproteins are phycocyanin and phycoerythrin [10]. Together, these three classes of pigments maximize light absorption by cyanobacteria across the entire visible spectrum. They exhibit a range of physicochemical properties and biological functions, making them a valuable subject of study in the field of biotechnology [11].

It is established that plants, microalgae, fungi and bacteria can be regarded as potential sources of natural pigments. However, plant-derived pigments have certain disadvantages, including dependence on seasonal raw materials (leaves, fruits, roots, etc.), sensitivity to extraction conditions, longer regeneration time, and difficulties in scaling up [12,13,14]. The production of mushroom-based pigments is costly due to fermentation, and they are prone to toxin production. Additionally, most bacteria-based pigments are still in the research and development stage [15]. It is crucial to consider the remarkable growth rate of cyanobacteria when contemplating various strategies for their management. Furthermore, their cultivation can be conducted on a continuous basis, which is regarded as the optimal system for large-scale biomass production, thereby rendering the process of natural pigment production cost-effective [16,17]. In contrast to heterotrophic bacteria, cyanobacteria are able to grow using only sunlight, carbon dioxide, water and minimal nutrients, thereby eliminating the cost of carbon sources and complex nutrient media. Furthermore, the alkalophilic nature of certain cyanobacterial strains, such as *Spirulina* sp., enables them to be cultivated with a reduced risk of contamination [18]. Sunlight is the most readily available and inexpensive resource on earth, and utilizing cyanobacteria to produce highly dispersed chemicals from solar energy offers a greener pathway for their biosynthesis process [19]. Furthermore, cyanobacteria exhibit higher biomass production rates than plants and have the capacity to convert up to 39% of solar energy into biomass, a figure that greatly surpasses the ≤0.25–3% achieved by agricultural crops such as maize and sugarcane [20]. The significant advantages of cyanobacteria over other natural pigment sources, coupled with the current trend of consumer awareness towards natural products, food safety and sustainable consumption, indicate a growing interest in cyanobacterial pigments as natural colorants in food products.

Particularly, the prospective medical applications of carotenoids, terpenoid pigments, in the treatment of cancer, respiratory, neurological or immunological degenerative pathologies are worthy of note. Additionally, they can serve as antibacterial, antiviral or anticholesterol agents and possess a high commercial value, being widely used as dietary supplements for humans and animals [21,22]. The principal carotenoids present in cyanobacteria are β-carotene, as well as the xanthophylls echinenone, zeaxanthin and specific carotenoids such as myxoxanthophyll [23]. Other carotenoids of biotechnological value, namely lutein, astaxanthin, canthaxanthin and β-cryptoxanthin, are found in low concentrations (depending on the species) and their role in the photosynthetic apparatus remains unclear [24]. A number of additional carotenoids have been identified in specific species, including synechoxanthin in *Synechococcus* [25], caloxanthin and nostoxanthin in *Nostoc* sp. [26], and alloxanthin in *Cryptomonas* sp. [27], and mutachrome has also been isolated from *Prochloron* sp. [28]. A distinctive class of carotenoid glycosides (e.g., oscillaxanthin, myxoxanthophyll) has been identified in select cyanobacterial species, including *Cylindrospermopsis* sp., *Thermosynechococcus* sp., *Synechocystis* sp., *Anabaena* sp. and *Nostoc* sp. [29,30].

Although cyanobacteria are not the organism of choice for industrial carotenoid production, optimizing their production and assessing their biological activity demonstrates that these organisms may indeed be potential candidates for future pigment production in a more environmentally friendly and sustainable bioprocessing approach [31]. In this context, the screening of cyanobacteria to identify new species containing valuable metabolites, including pigments and the optimization of their production are crucial steps in the exploitation of the full potential of these interesting targets.

The present study is concerned with the pigment composition of cyanobacterial strains isolated from hot springs, with the objective of identifying producers of valuable pigments for biotechnological applications.

## 2. Results

### 2.1. Isolation and Identification of Cyanobacterial Strains from Hot Springs

Water samples were collected from four wells numbered 1587, 3422, 141-D and 1478 in different fields in the Uygur district (Figure S1). The temperature of the hot springs during the sampling period was 38–50 °C, and the pH was recorded in the range of 7–8, indicating an alkaline environment. According to the data, the composition of water in the investigated wells No. 1587 (geo-coordinates: 43.63709536470939, 80.05503078936722) and No. 3422 (geo-coordinates: 43.637083272623734, 80.05405526078577) was characterized as slightly mineralized (up to 0.43 g dm^−3^), slightly alkaline (pH 8.1–8.4), nitric (up to 95%), hyperthermal with complex chloride–sulphate–hydrocarbonate–calcium–sodium composition, containing an increased concentration of silicic acid (39.0–40.0 mg dm^−3^). The mineral waters from wells No. 141-D (geo-coordinates: 43.65516785916136, 80.16010043702464) and No. 1478 (geo-coordinates: 43.66404336640531, 80.17233815840281) of the Karadala deposit are therapeutic, nitric, slightly mineralized, slightly alkaline and identified as thermal waters of complex sulphate–chloride–hydrocarbonate (No. 141-D) and hydrocarbonate–sulphate–sulphate–chloride (No. 1478) sodium composition [32].

The algoflora composition and structure of samples from thermal springs were determined using the cup culture method. The most active and dominant species were identified by direct microscopy, as well as by their viability and development in the culture fluids. A total of 22 species of cyanobacteria and 16 species of microalgae were detected in the studied water samples. The following dominant microalgal genera were found in all the studied sites *Chlorella*, *Dunaliella*, *Euglena*, *Scenedesmus*, as well as genera of cyanobacteria—*Synecphococcus*, *Phormidium*, *Nostoc*, *Oscilatoria*, *Anabaena*, etc. Cyanobacteria include 22 species represented by 15 intraspecific taxa and belong to five orders (*Chroococcacales*, *Spirulinales*, *Nostocales*, *Oscillatoriales*, *Synechococcales*). Among the five orders, the species diversity is dominated by *Oscillatoriales*—13 or 6.58%, followed by *Nostocales*—4 or 3.70% and *Chroococcacales*—5 or 3.04%. The order *Oscillatoriales* is represented by 6 families and 11 genera. Among them, the family *Oscillatoriaceae*—5 and the genus *Phormidium*—3 occupy the leading position in terms of richness of forms. Dominant species were cultivated in nutrient media for further isolation and purification from associated microflora.

From these, four axenic cyanobacterial cultures were isolated and labeled with the name of the culture and the serial number of the collected sample: *Oscillatoria subbrevis* CZS 2201, *Phormidium ambiguum* CZS 2205, *Synechococcus* sp. CZS 2204, *Nostoc calcicola* TSZ 2203.

**Figure 1:**
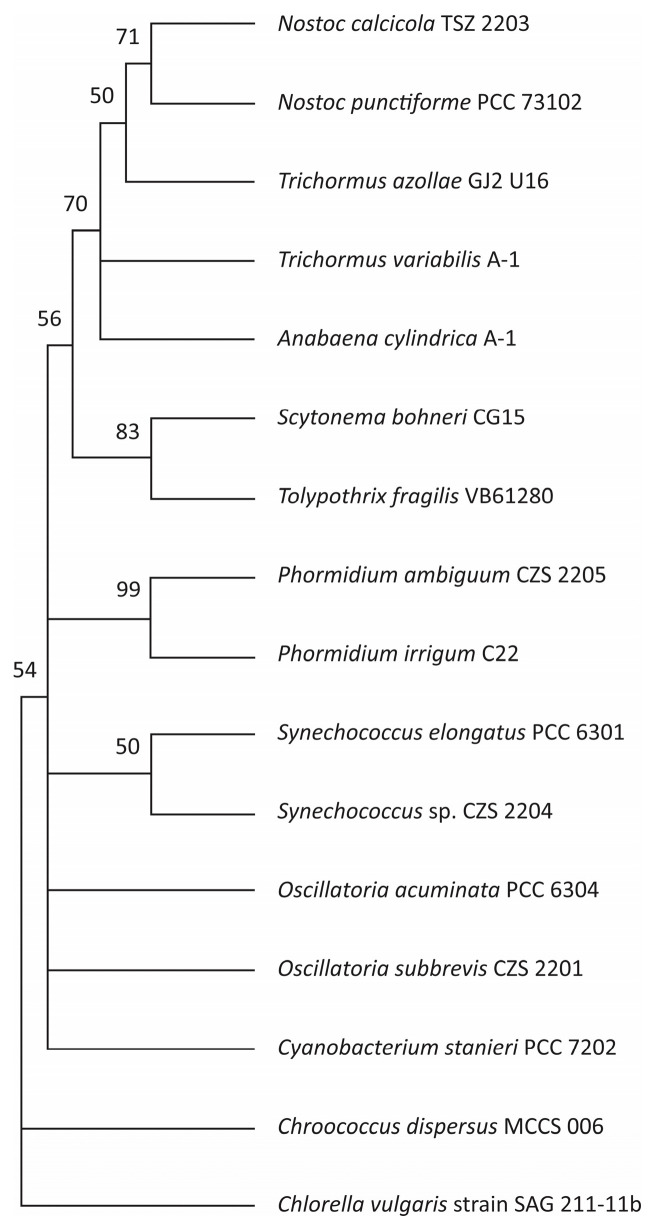
Phylogenetic tree of the isolated cyanobacterial strains and neighboring species.

The novel isolated strains of *Oscillatoria subbrevis* CZS 2201 (OQ627016), *Phormidium ambiguum* CZS 2205 (OQ646791), *Synechococcus* sp. CZS 2204 (OQ627024) and *Nostoc calcicola* TSZ 2203 (OQ627023) were amplified using universal 16s rRNA primers. *Chlorella vulgaris* SAG 211-11b strain was used as an external group. Figure 1 shows the phylogenetic relationships of the adjacent strains and the tree topology, which reflects the basis of the sequential taxon comparison. The phylogenetic tree consists of four main clusters and one group, each containing mainly different representatives of *Synechococcus*, *Oscillatoria*, *Phormidium* and *Nostoc* species. *Oscillatoria subbrevis* CZS 2201 strain showed similarities with *Oscillatoria subbrevis* PCC7202 (94.33%) and *Cyanobacterium stanieri* PCC7202 (92%). Although we observed phylogenetic proximity of *Oscillatoria subbrevis* CZS 2201 strain to both strains, it was found to be morphologically related to the *Oscillatoria* type. The *Synechococcus* sp. CZS 2204 strain showed affinity to the species of *Synechococcus elongatus* RCC 6301 (93.17%). The 1444 nucleotide chains were used for the phylogenetic analysis of *Phormidium ambiguum* CZS 2205 strain. The *Phormidium ambiguum* CZS 2205 strain was found to be 94.00% closely related to the *Phormidium irriguum* C22 strain. In addition, *Nostoc calcicola* TSZ 2203 strain (92.46%) and *Nostoc punctiforme* PCC 73102 strain (90.92%) also showed genetic similarity to the strain under investigation.

### 2.2. Growth and Morphological Characterization of Selected Cyanobacterial Strains

Biomass yield, growth rate and photosynthetic activity are the main indicators for screening the productivity of cyanobacteria. Cyanobacterial isolates of the genera *Oscillatoria*, *Phormidium*, *Synechococcus*, *Nostoc* were selected based on comparative productivity analysis, which includes growth rate and dry mass. The initial optical density was 0.2 at 720 nm in all cases and monitored daily. Dry mass was measured every 3 days.

**Figure 2:**
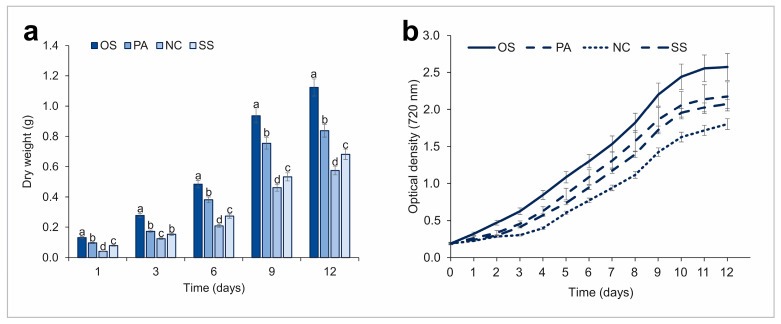
Growth profile of new isolated cyanobacterial strains. Note: (**a**)—dry biomass, (**b**)—growth rate. Note: OS—*Oscillatoria subbrevis* CZS 2201, PA—*Phormidium ambiguum* CZS 2205, SS –*Synechococcus* sp. CZS 2204, NC—*Nostoc calcicola* TSZ 2203. Note: a,b,c,d mean statistical significance (*p* < 0.001).

According to the graph (Figure 2a), *Oscillatoria* and *Phormidium* strains had the highest dry biomass accumulation values of 1.12 and 0.84 g L^−1^, respectively, on the 12th day of cultivation. The lowest dry mass values were found for *Nostoc* with 0.57 g L^−1^. As shown in Figure 2b, the highest cell growth rate was observed in *Oscillatoria* (2.58) and the lowest in *Nostoc* (1.81) on 12th day of cultivation. And *Phormidium* and *Synechococcus* cultures showed similar growth values approximately at 2.18 and 2.08, respectively. Thus, as a result of the growth dynamics study of the four isolated cyanobacterial cultures, *Oscillatoria* showed high growth rates and biomass yield, indicating its high productivity.

**Figure 3:**
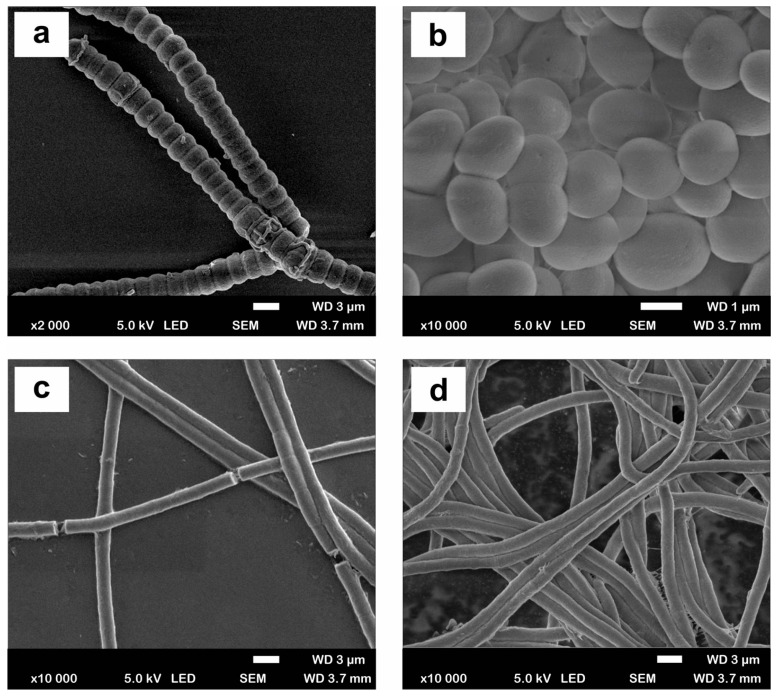
Scanning electron microscopy (SEM) micrographs of (**a**) *Nostoc calcicola* TSZ 2203, (**b**) *Synechococcus* sp. CZS 2204, (**c**) *Phormidium ambiguum* CZS 2205, (**d**) *Oscillatoria subbrevis* CZS 2201.

In terms of basic morphological characteristics, *Nostoc calcicola*, a member of the family *Nostocaceae*, is a filamentous cyanobacterium with intercalary heterocysts (specific atmospheric N_2_-binding cells) and akinetes cells formed centrifugally in a row between heterocysts (Figure 3a and Figure S2a). The width of the heterocysts ranged from 1.54 to 3.37 μm and the length from 3.21 to 6.19 μm. The shape of the heterocysts varied from ovoid or spherical to ellipsoidal. The heterocysts were both terminal and intercalated.

The *Synechococcus* strain, from the family *Synechococcaceae*, has round or binary cells with rounded ends; mature cells ranged in size from 0.8 to 1.2 µm wide and 2.4 to 3.1 µm length (Figure 3b and Figure S2b). Unique polar divisions with the formation of spherical (globular) cells were observed. The width and shape of the constriction region were similar.

*Phormidium ambiguum*, from the family *Oscillatoriaceae*, is a filamentous type of cyanobacterium (Figure 3c and Figure S2c) that does not fix nitrogen because it lacks heterocytes. The filaments were unbranched but divided into short fragments. The ends of the filaments were straight without twisting. The filaments were composed of cylindrical cells whose length slightly exceeded their thickness (1.84 μm).

The next strain of the family *Oscillatoriaceae*, identified as *Oscillatoria subbrevis*, was also a filamentous form without heterocysts (Figure 3d and Figure S2d). Trichomes were solitary, almost straight, not retracted at the apex, not overdrawn at the transverse walls; cells were not granulated at the transverse walls, cell length varied between 1.5–3.1 µm and width between 6.5 and 7.1 µm; the terminal cell was rounded; and calyptra was absent.

### 2.3. Pigments Analysis of Cyanobacterial Strains

The current study examined the pigment profile, in terms of chlorophyll *a* and total carotenoids content of selected cyanobacterial cells. A trend towards increased production of chlorophyll *a* and total carotenoids was observed up to 9 days of cultivation, after which a decrease in both chlorophyll *a* and total carotenoids was observed under controlled conditions where light, nutrient availability and other environmental conditions were not limiting factors.

As illustrated in Table 1, during the initial phase (1–3 days), the pigment content of all species is typically low, as the cells adapt to the novel environment and are not actively proliferating. During the exponential phase (3–9 days), the content of chlorophyll-*a* and carotenoids increases rapidly, as pigment synthesis is closely related to biomass increase. This is because pigments are essential for photosynthesis and energy production during this phase of rapid growth. During the stationary phase (11–12 days), the growth rate decelerates as nutrients become scarce and cell numbers reach a plateau. At this juncture, pigment concentrations may stabilize or undergo a slight decline as cells begin to experience nutrient limitation. However, some cyanobacteria may continue to accumulate certain pigments, such as carotenoids, as a response to stressful environmental conditions.

**Figure 4:**
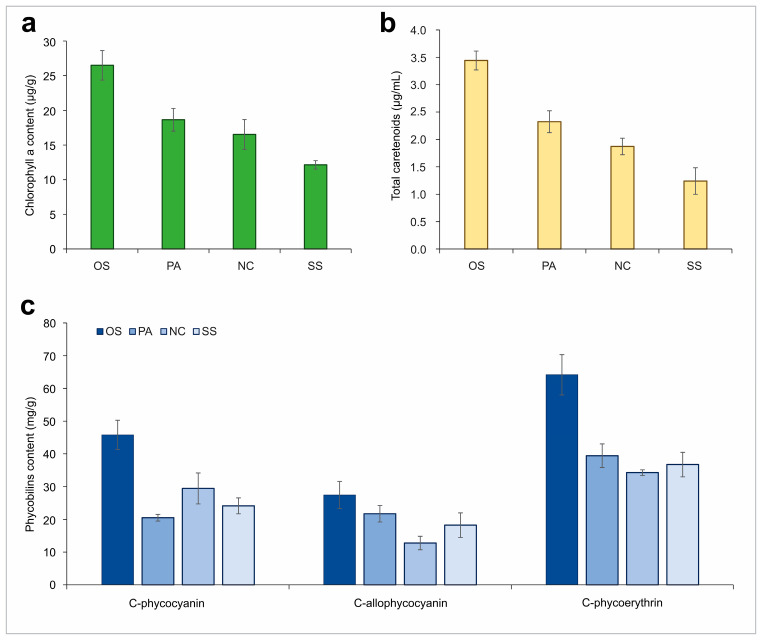
Pigments profile of cyanobacterial monocultures: (**a**)—chlorophyll a; (**b**)—total carotenoids content; (**c**)—phycobiliprotein content. Note: OS—*Oscillatoria subbrevis* CZS 2201, PA—*Phormidium ambiguum* CZS 2205, SS—*Synechococcus* sp. CZS 2204, NC—*Nostoc calcicola* TSZ 2203.

The analysis revealed that the maximum quantity of chlorophyll *a* and total carotenoids was observed in *Oscillatoria subbrevis* CZS 2201 (26.49 and 3.44 µg mL^−1^), followed by *Phormidium ambiguum* CZS 2205 (18.64 and 2.32 µg mL^−1^), whereas a minimum chlorophyll *a* and total carotenoids amount was detected in *Synechococcus* sp. CZS 2204 (12.13 and 1.24 µg mL^−1^), respectively. Chlorophyll *a* and total carotenoids in *Nostoc calcicola* TSZ 2203 valued for 16.52 and 1.87 µg mL^−1^ (Figure 4a,b).

In addition to photoprotective pigments, other accessory pigments—phycobilins, a group of three pigments, C-phycocyanin, C-allophycocyanin and C-phycoerythrin were measured. The maximum value of C-phycocyanin was detected in *Oscillatoria subbrevis* CZS 2201 (45.81 mg g^−1^), followed by *Nostoc calcicola* TSZ 2203 (29.44 mg g^−1^) and *Synechococcus* sp. CZS 2204 (24.13 mg g^−1^), whereas a minimum C-phycocyanin content was measured for *Phormidium ambiguum* CZS 2205 (20.5 mg g^−1^). Regarding C-phycoerythrin, the high content was observed also for *Oscillatoria subbrevis* CZS 2201 cells with (64.17 mg g^−1^) and the lowest value was in *Nostoc calcicola* TSZ 2203 (34.27 mg g^−1^). Similarly, C-allophycocyanin content was maximum in *Oscillatoria subbrevis* CZS 2201 about at 27.45 mg g^−1^, and the minimum in *Nostoc calcicola* TSZ 2203 at 12.78 mg g^−1^ (Figure 4c).

Based on the obtained results, *Oscillatoria subbrevis* CZS 2201 showed high contents for all examined pigments and was selected for further identification of carotenoid composition.

### 2.4. Carotenoid Composition of Oscillatoria subbrevis CZS 2201

**Figure 5:**
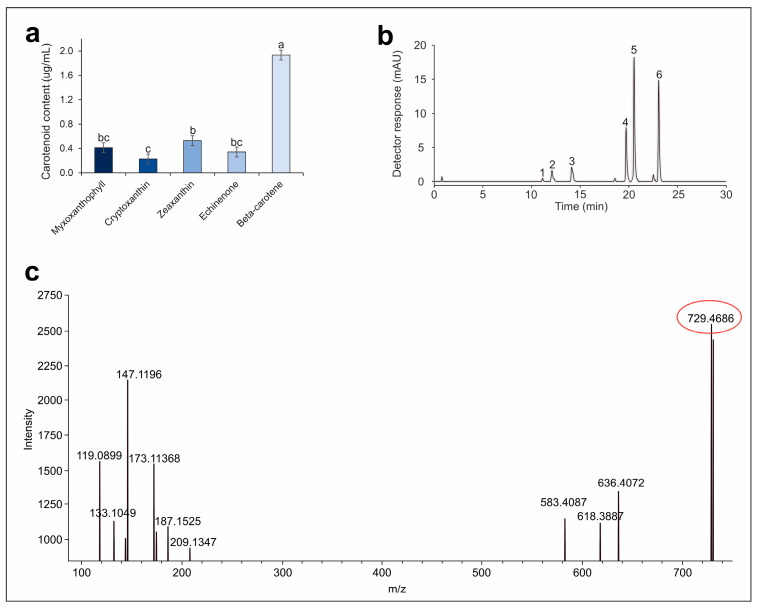
Pigment analysis of *Oscillatoria subbrevis* CZS 2201: (**a**)—carotenoid composition; (**b**)—chromatographic profile of pigments: 1—myxoxanthophyll 1, 2—myxoxanthophyll 2, 3—zeaxanthin, 4—echinenone, 5—chlorophyll *a*, 6—β-carotene; (**c**)—ESI-HR/MS spectrum of myxoxanthophyll. Note: a,b,c mean statistical significance (*p* < 0.001), bc means statistical significance (*p* < 0.01).

In this study, the carotenoid composition of the thermophilic filamentous cyanobacterium *Oscillatoria subbrevis* CZS 2201, grown photoautotrophically under diazotrophic conditions at 32 °C, was also examined. According to the obtained results, carotenoids such as myxoxanthophyll, zeaxanthin, echinenone and β-carotene were identified. The predominant form among the carotenoids was β-carotene (1.94 µg mL^−1^), while the accumulation of zeaxanthin and myxoxanthophyll was relatively high (0.53 and 0.41 µg mL^−1^, respectively) in comparison to echinenone and cryptoxanthin (0.34 and 0.23 µg mL^−1^, respectively) (Figure 5a,b).

The chemical identity of the isolated target compound was established using a Dionex UltiMate 3000 HPLC system (Thermo Scientific, Sunnyvale, CA, USA) connected to a high-resolution tandem mass spectrometry (HRMS/MS) detector with electrospray ionization (ESI) source (Impact HD mass spectrometer, Bruker, Billerica, MA, USA) (HPLC-ESI-HRMS/MS). The identification of the target compound was confirmed in comparison with the published literature data [33]. The ESI-HRMS spectrum of the target compound peak indicated a molecular formula, consistent with C_46_H_64_O_7_, accompanied by a cation-radical molecular ion [M]+ at *m*/*z* 729.4686 (Figure 5c). The ESI-HRMS/MS fragmentation of the cation radical molecular ion gave a fragment ion at *m*/*z* 581.3995, corresponding to the carotenoid moiety formed after cleavage of the sugar moiety (C_6_H_11_O_4_), which are in agreement with the fragmentation pattern of (all-trans)-myxoxanthophyll as 2′-(3-O-methyl-α-L-fucoside).

## 3. Discussion

Thermophilic cyanobacteria are of scientific interest due to their analogy with ancient life forms on Earth and are a valuable source of thermostable biomolecules [33]. They have evolved a variety of adaptations to withstand extreme habitat conditions and exhibit a high degree of thermotolerance, enabling them to survive and function at elevated temperatures. The lipid composition of the cell membrane of thermophilic cyanobacteria is adapted to withstand high temperatures and contains a higher proportion of saturated fatty acids. This increases the rigidity and stability of membranes, prevents their denaturation and promotes the re-clotting of damaged proteins [34]. Furthermore, they produce a class of proteins, designated as heat shock proteins in response to elevated temperatures, which assist in stabilizing cellular components [35]. Additionally, thermophilic cyanobacteria frequently possess distinctive pigment compositions that serve to safeguard them from high levels of light and ultraviolet radiation in hot habitats. They are capable of synthesizing specific carotenoids and mycosporine-like amino acids, which function as photoprotective compounds [36,37]. From this perspective, the isolation and characterization of cyanobacteria from hot springs is of practical importance, as the isolated culture may have biotechnological potential to produce bioactive molecules and thermostable enzymes. Considering the potential of cyanobacteria in pigment production, the present study investigated four novel axenic cyanobacterial strains isolated from Chundzha hot springs, namely *Oscillatoria subbrevis* CZS 2201, *Phormidium ambiguum* CZS 2205, *Synechococcus* sp. CZS 2204 and *Nostoc calcicola* TSZ 2203. Furthermore, comparative analyses were conducted on the productivity of the strains, as well as on the content of chlorophyll a, total carotenoids and phycobiliproteins, with the aim of identifying potential pigment producers.

Carotenoids are widely described as bioactive compounds, including antioxidants, anti-inflammatory agents, anti-tumor promoters and antimicrobial agents, which can be used in animal feed as color enhancers and in cosmetics as antioxidants and anti-aging components [38]. The growing utilization of carotenoids in food, feed, pharmaceutical and cosmetic industries has led to an increased demand for natural carotenoids in the market. It is anticipated that the global market value of natural carotenoids will reach USD 2 billion by 2022 [3]. However, the majority of carotenoids used in industry are derived from microalgae and plants. Consequently, there is a paucity of studies concerning cyanobacterial carotenoids and their activity. A substantial body of literature exists on the determination of pigment content in the biomass of green and other higher algae [39]. Their distinctive morphological and metabolic characteristics position them as the most promising candidates for the production of natural dyes [40]. However, cyanobacteria are also the most efficient photosynthetic organisms, probably because they have a very fast response and adaptation to environmental stimuli, which no other group of living things has been observed to possess [41]. Furthermore, cyanobacteria possess a distinctive and adaptable pigment composition, enabling them to absorb light across a broad spectrum and to store considerable quantities of light energy [42]. In a recent study, Pagels et al. [43] reported that cyanobacteria of the genus *Cyanobium* are rich in not only β-carotene but also pigments such as echinenone, lutein and zeaxanthin, which possess a range of properties, including photoprotective, anti-obesity, anti-inflammatory, neuroprotective, antidiabetic, antioxidant and anticancer effects. A similar outcome was observed in a study conducted by Nayak et al. [44], wherein a set of 24 heterocystic cyanobacterial strains belonging to the genus *Anabaena*, isolated from diverse geographical locations in India, were examined for their pigment profile, revealing notable discrepancies in chlorophyll and carotenoid accumulation.

Furthermore, the photosynthetic apparatus of cyanobacteria contains several water-soluble phycobiliproteins organized in phycobilisomes. These molecules act as free radical scavengers, thereby increasing their use in the pharmaceutical industry and as fluorochromes in biomedical research [45]. Phycobiliproteins are also reported to be effective anticancer, neuroprotective, anti-inflammatory, anti-allergic and hepatoprotective agents [46]. Ferraro et al. [47] carried out the purification of C-phycocyanin from the extremophilic species of Galdieri. The study by Liang et al. [48] showed that C-phycocyanin synthesized by *Synechococcus* sp. was more stable than C-phycocyanin synthesized by *Spirulina* species. In another study, *Galdieria phlegrea* strain isolated from different geographical areas were also tested for their thermostable C-phycocyanin during autotrophic and heterotrophic cultivation [48].

In this study, a screening of cyanobacteria strains was conducted, revealing that the maximum chlorophyll a content was 26.49 µg mL^−1^ and the maximum production of total carotenoid was 3.44 µg·mL^−1^ in *Oscillatoria subbrevis* CZS 2201 cells. Tabassum et al. [49] conducted a comparative study on four strains of four different genera of blue-green algae, including *Oscillatoria*, *Lyngbya*, *Anabaena* and *Microchaete*, and reported a dry weight of chlorophyll *a* (506 μg g^−1^), carotenoids (58 μg g^−1^), C-phycocyanin (1420 μg g^−1^), allophycocyanin (1058 μg g^−1^) and C-phycoerythrin (422 μg g^−1^) in *Oscillatoria* under controlled conditions [49]. Another study of Jeevanantham et al. [50] demonstrated biochemical characteristics of five marine cyanobacteria species, where the maximum quantity of chlorophyll a was observed in *Synechococcus aeruginosus* (58.14 mg mL^−1^), while the highest total carotenoid content was recorded in *Synechococcus elongatus* (7.49 μg g^−1^).

The maximum quantities of C-phycocyanin, C-allophycocyanin and C-phycoerythrin produced by *Oscillatoria subbrevis* CZS 2201 were also determined to be 45.81, 27.45 and 64.17 mg g^−1^, respectively. Similar data were obtained in the study of Sarmah and Rout [51], which presented the biochemical composition of five cyanobacteria isolated from submerged polythene surface in domestic sewage water, where the maximum growth rate and the maximum total phycobiliproteins has been shown by *Oscillatoria subbrevis* (0.158 μ d^−1^ and 81.4 μg mL^−1^, respectively). Another study demonstrated the biomass and biomolecule profiling of *Oscillatoria subbrevis* and *Oscillatoria sancta*, with growth rates of 0.107 μ d^−1^ and 0.140 μ d^−1^, respectively. In addition, the maximum C-phycocyanin, C-allophycocyanin and C-phycoerythrin produced by *Oscillatoria subbrevis* were 0.007 μg mL^−1^, 0.002 μg mL^−1^ and 0.04 μg mL^−1^, respectively, after 20 days of batch culturing [52]. The most recent study on *Oscillatoria* spp. strain demonstrated considerable variability in chlorophyll *a* and carotenoid contents, with values ranging from 12.67 to 22.72 and 1.0 to 1.4 µg mL^−1^, respectively. Additionally, phycobiliprotein contents exhibited a broader range, spanning from 87.39 to 121.42 µg mL^−1^ [53]. The data presented will contribute to the screening and characterization of cyanobacteria of the genus *Oscillatoria* in terms of pigment, with a view to their use in research and various commercial applications.

Additionally, the findings of the current study indicate a considerable concentration of carotenoids within the cells of the *Oscillatoria subbrevis* 2201. The predominant form among the product carotenoids was β-carotene (1.94 µg mL^−1^), while the accumulation of zeaxanthin and myxoxanthophyll was relatively high (0.53 and 0.41 µg mL^−1^, respectively) in comparison to echinenone and cryptoxanthin (0.34 and 0.23 µg mL^−1^, respectively). Furthermore, the carotenoid composition in the cells of the thermophilic culture *Oscillatoria* can be modulated by growth temperature. At suboptimal temperatures, β-carotene is the dominant carotenoid, whereas increasing the temperature leads to the accumulation of ketocarotenoids, including myxoxanthophyll. Myxoxanthophyll [3R, 2′S)-2′-(2,4-di-O-methyl-α-L-fucoside)-3′,4′-didehydro-1′,2′-dihydro-β,ψ-carotene-3,1′-diol] is a xanthophyll glycoside characterized by a unique glycosidic bond, found in cyanobacteria and some non-photosynthetic bacteria, but not in eukaryotic algae. Its structure has been identified as a mixed carotenoid glycoside, where the dominant sugar moiety is rhamnose, and hexose is a minor component [54]. According to the literature data, several myxol glycosides with fucose derivatives have been identified, including myxol 2′-(3-O-methyl-α-l-fucoside) from *Oscillatoria bornetii* [55], non-methylated myxol 2′-α-l-fucoside from *Oscillatoria limnetica* [56], and myxol 2′-dimethylfucoside from the strain *Synechocystis* sp. PCC 6803 [54].

The presence of a sugar residue (pentose or, less frequently, hexose) in the structure of myxoxanthophyll renders the molecule highly amphiphilic in comparison to other xanthophylls, which exhibit predominantly hydrophobic properties. The presence of a terpenoid aglycone with a conjugated system of 11 double bonds, similar to that present in other xanthophylls such as zeaxanthin or lutein, indicates that these compounds possess high reactivity with active oxygen species [57]. Studies conducted in recent years by several independent research groups have demonstrated that myxoxanthophyll possesses significantly higher antioxidant potential than β-carotene [57]. In the study of Nováková et al. [58] 20 mg of myxoxanthophyll was yielded (95% purity) from 280 mg extract of *Synechocystis salina*, and the target pigment showed a weak antioxidant and tyrosinase inhibitory effect and exhibited immune-stimulating properties by activating human granulocytes. In addition, glycoside xanthophylls are of greater economic interest than β-carotene due to their high coloring power, which can be applied as a natural coloring agent and food additive in various products such as juices, beverages, dairy products, confectionery and gelatin [59].

## 4. Materials and Methods

### 4.1. Sampling and Isolation of Cyanobacterial Strains

This study focused on cyanobacterial cultures isolated from hot springs in the Chundzha settlement of the Uygur district, situated approximately 250 km (155 mi) from Almaty city, Kazakhstan. Water samples from these sites were collected in pre-sterilized 1 L plastic bottles. The water samples were filtered through 0.45 µm pore diameter membranes and transported to the laboratory, where they were stored at 4 °C in a refrigerator until further processing. Biological mats, concretions and sediments were randomly collected with sterile forceps and spatula from the sampling sites and placed in sterile glass containers. Water samples, to check for the presence of planktonic strains of cyanobacteria, were collected in sterile glass vials and tubes. Sampling was conducted between 10 and 25 October 2021. The temperature and pH of the water at the site were measured using a thermometer and a digital pH meter (HM Digital Inc., Los Angeles, CA, USA), respectively.

To obtain enrichment cultures, algae were grown in glass 250 mL Erlenmeyer flasks containing various nutrient medium at a temperature of 24 ± 3 °C, with periodic illumination at intensity of 54–90 μmol m^−2^ s^−1^ for FLUORA-type fluorescent lamps (LEDVANCE GmbH, Munich, Germany), a light cycle of 16:8 (day:night). Subsequently, the cultures were observed under a microscope regularly, with reseeding conducted at intervals of approximately every few days. These reseedings were performed by repeated serial dilution on selective artificial nutrient medium with regulation of acid-alkaline conditions. The isolation and purification of axenic cyanobacterial cultures were conducted using microbiological, mechanical and chemical methods, including stroke sowing, micropipette isolation, and multiple reseeding on solid (agar) media [60]. The morphological characteristics of the isolated cyanobacterial cultures were studied using a MicroOptix series microscope (West Medica, Wiener Neudorf, Austria) with image display on a monitor, while species composition was determined using existing identifiers [61,62,63,64,65].

### 4.2. Molecular Identification of Cyanobacterial Isolates

The molecular identification of microorganisms was conducted through the utilization of molecular genetic techniques, specifically through the sequencing of a conserved DNA or ribosomal RNA locus. Genomic DNA of varying lengths (>1000 bp was isolated using the protocol outlined in [66]. The 16S RNA gene was amplified from cyanobacterial genomic DNA using universal primers 27F (5′-AGAGAGTTTGATCCTGGCTCAG-3′) and 1492R (5′-TACGGTTACCTTGTTACGACTT-3′). The DNA sequences were compared by BLAST, version 2.11.0 (accessed on 15 June 2024, https://blast.ncbi.nlm.nih.gov/Blast.cgi) analysis to identify homologous taxa available on the NCBI DNA database [67] and were multiple-aligned using the MUSCLE program. The phylogenetic tree was constructed using Molecular Genetics Analysis (MEGA) 6.06 software [68], based on evolutionary distances, and calculated using the Neighbor-Joining method based on the Kimura-2-Parameter algorithm. The statistical evaluation of the tree topologies was conducted through a bootstrap analysis of 1000< repeated samples.

### 4.3. Growth Profile of Selected Cyanobacterial Cultures

Axenic cultures of the studied cyanobacteria were grown in a laboratory cultivator in 500 mL glass vessels containing 200 mL of modified Zarrouk Medium (g/L: 1.0 NaCl, 0.04 CaCl_2_·2H_2_O, 2.5 KNO_3_, 0.01 FeSO_4_·7H_2_O, 0.08 EDTA (Na), 1.0 K_2_SO_4_, 0.2 MgSO_4_·7H_2_O, 16.8 NaHCO_3_, 0.5 K_2_PO_4_) [69] and BG11 (g/L: 1.5 NaNO_3_, 0.036 CaCl_2_·2H_2_O, 0.012 Ferric ammonium citrate, 0.001 EDTA-Na_2_·2H_2_O, 0.04 K_2_HPO_4_, 0.075 MgSO_4_·7H_2_O, 0.02 Na_2_CO_3_ and 1 mL trace metals: mg/L: 2.86 H_3_BO_3_, 1.81 MnCl_2_·4H_2_O, 0.222 ZnSO_4_·7H_2_O, 0.39 Na_2_MoO_4_·2H_2_O, 0.079 CuSO_4_·5H_2_O, 0.049 Co(NO_3_)_2_·6H_2_O) [70] at 28–32 °C for 12 days until reaching stationary phase, where they were aerated under artificial light with a light intensity of 53–62 photons μmol m^−2^ s^−1^ and a sterile gas/air mixture enriched with 1% CO_2_ *v*/*v* (Table S1). To determine the growth dynamics of the isolated cultures, 5 mL samples were collected aseptically every 24 h and analyzed spectrophotometrically at a wavelength of λ = 720 nm to monitor the growth of cyanobacterial cultures using a spectrophotometer (SPECORD^®^ S-600, Analytik Jena, Jena, Germany). The growth rate coefficient of cyanobacteria was defined as the difference between the maximum and initial density of the cell suspension [71]. The dry mass (DM) was analyzed every 3 days. Cyanobacterial suspensions were collected during the stationary growth phase by centrifugation (5804 R, Eppendorf, Hamburg, Germany) for 10 min at 4500 rpm. The separated cells were washed three times with distilled water and the number of fresh cell pellets was determined. The DM was recorded after drying the pellets in an oven at 60 °C until a constant weight was reached [72].

### 4.4. Scanning Electron Microscopy (SEM)

Cyanobacterial samples were fixed in 2.5% glutaraldehyde in Millonig’s phosphate buffer 0.2 M, pH 7.2 (Electron Microscopy Sciences, Hatfield, PA, USA) for 24 h. The specimens were washed thrice with the same buffer before being postfixed with 1% osmium tetroxide in MPB for 2 h. Finally, they were washed three times with MPB before being dehydrated in a graded acetone series. The dry samples were placed on a gummed standard SEM aluminum stub and were then gold-coated for 2 min in a sputter coater (Q150T, Quorum, Germany). Coated samples were examined directly under the SEM (Carl Zeiss AURIGA® CrossBeam®, Oberkochen, Germany) [73].

### 4.5. Pigment Analysis

#### 4.5.1. Extraction and Quantification of Phycobiliproteins

For the extraction of phycobiliprotein, a known volume of homogenized culture [74] was taken and centrifuged at 4000 rpm for 10 min at room temperature. The cell pellet was washed 2–3 times with distilled water and suspended in 20 mM acetate buffer (pH 5.1) containing 40 mM NaCl and 0.02 M sodium azide. This was followed by repeated freeze–thaw until the supernatant collected became colorless [75]. The collected supernatant was used for the estimation of phycobiliproteins expressed in mg/mL using following equation [76,77]:

Phycocyanin (PC) = [A615 − (0.474 × A652)]/5.34,(1)

Allophycocyanin (APC) = [A652 − (0.208 × A615)]/5.09,(2)

Phycoerythrin (PE) = [A662 − (2.41 × PC − 0.849 × APC)]/9.52,(3)

#### 4.5.2. Extraction and Quantification of Chlorophyll a, Total Carotenoids

Biomass of cyanobacteria (20 mg) was extracted with 5 mL of 100% methanol in an ultrasonic bath for 30 min; resulting extracts were combined and further centrifuged (1350 rpm, 10 min) to separate insoluble particles. The extraction procedure was repeated until the supernatant became colorless. The extract was partitioned in a mixture of petroleum ether/diethyl ether [1:1 (*v*/*v*)], and methanol was removed by washing with distilled water. The extract was concentrated in a rotary evaporator (T < 30 °C), flushed with N_2_ and kept at 37 °C in the dark. The determination of the chlorophyll a and total carotenoids’ concentration in the extract was determined spectrophotometrically [78] and calculated according to following equation [79]:

Chlorophyll a (Chl a) =16.72 × A665.2 − 9.16 × A652.4,(4)

Total carotenoid content (Cars) = [1000 × A470 − 1.63 × Chl a]/221,(5)

#### 4.5.3. HPLC/MS Analysis of Pigments

For the determination of cyanobacterial pigment content was used UHPLC (Thermo Scientific, Sunnyvale, CA, USA) equipped with a diode array detector (DAD) and Impact HD high resolution mass spectrometer (Bruker, Billerica, MA, USA) calibrated with sodium formate clusters and LockMass. Obtained molecular peaks and fragments are calculated using Smart Formula in Bruker Compass DataAnalysis software (version 4.2). Pigments are separated using a modified method of [58] on the Luna C8 (2) 100Å column (100 × 4.6 mm, 3 μm, Phenomenex, Taipei City, Taiwan) thermostated to 30 °C, with binary solvent system (0 min 100% A, 20 min 100% B, 25 min 100% B, 27 min 100% A, 30 min 100% A, where A is 80% methanol and B is 100% methanol). The solvent flow rate 0.8 mL min^−1^ and injection volume 20 μL. The identification of the carotenoids is performed, considering the combination of the following parameters: elution order on the reverse phase column, UV–vis spectrum features (maximum absorption wavelength (λmax), spectral fine structure (%III/II) and cis-peak intensity (%Ab/AII)), MS and MS/MS spectra characteristics (protonated molecule ([M + H]^+^) and MS/MS fragments), compared with data available in the literature [80,81,82,83,84].

### 4.6. Statistical Analysis

All experiments were conducted with 3–5 replicates. The results were evaluated by Analysis of Variance (ANOVA) to determine a statistically significant difference. Significance was declared at *p* < 0.05. The graphs represent the arithmetic means (M) and standard deviations (SD) [85].

## 5. Conclusions

A comparative productivity analysis revealed that *Oscillatoria subbrevis* CZS 2201 exhibited a high growth rate and biomass yield, as well as elevated levels of chlorophyll *a*, total carotenoids and phycobiliproteins. Consequently, this organism was selected for further biochemical studies on carotenoid composition, with the objective of identifying potential pigment producers. The major carotenoids detected in the cyanobacterium *Oscillatoria subbrevis* CZS 2201 were β-carotene, as well as the xanthophylls echinenone, zeaxanthin and specific carotenoids such as myxoxanthophyll. The predominant form among the product carotenoids was β-carotene, while the accumulation of zeaxanthin and myxoxanthophyll was relatively high in comparison to echineenone and cryptoxanthin. Therefore, the recently isolated strain of the cyanobacterium *Oscillatoria subbrevis* 2201 is a promising producer of carotenoid derivatives, including myxoxanthophyll, and also exhibits the capacity for rapid biomass reproduction and high productivity.

## Figures and Tables

**Table 1:** Content of chlorophyll *a* and total carotenoids in selected cyanobacterial cells during the period of cultivation, µg mL^−1^.

Period of Cultivation	OS		PA		SS		NC	
	Chl *a*	Total Cars	Chl *a*	Total Cars	Chl *a*	Total Cars	Chl *a*	Total Cars
1-day	5.22	0.475	4.48	0.314	2.36	0.291	3.63	0.308
3-day	13.62	1.301	9.46	0.691	5.64	0.488	8.25	0.511
6-day	21.14	1.873	15.33	1.212	9.32	0.716	11.96	0.893
9-day	26.49	3.442	18.64	2.324	12.13	1.241	16.52	1.872
12-day	19.23	3.121	14.21	1.977	10.65	1.025	13.24	1.417

Note: OS—Oscillatoria subbrevis CZS 2201, PA—Phormidium ambiguum CZS 2205, SS—Synechococcus sp. CZS 2204, NC—Nostoc calcicola TSZ 2203.

## Data Availability

The original contributions presented in the study are included in the article/Supplementary Material, further inquiries can be directed to the corresponding author.

## Supplementary Materials

The following supporting information can be downloaded at: https://www.mdpi.com/article/10.3390/plants13212951/s1, Figure S1. Geographical illustration of sampling points from Chundzha hot springs (GeoCoordinates: 43°32’14.9’’N 79°27’56.0’’ E). Figure S2. Light microscopy micrographs of (a) *Nostoc calcicola* TSZ 2203, (b) Synechococcus sp. CZS 2204, (c) *Phormidium ambiguum* CZS 2205, (d) *Oscillatoria subbrevis* CZS 2201. Table S1. The taxonomic position and the culture conditions of the maintenance of novel cyanobacterial isolates.
