# Supplementary material for: Isolation, Identification and Pigment Analysis of Novel Cyanobacterial Strains from Thermal Springs
Source: Plants (Basel). 2024 Oct 22;13(21):2951. doi: 10.3390/plants13212951 (PMC11547633; doi:10.3390/plants13212951)
Supplement: Supplementary file 1 [file plants-13-02951-s001.zip › plants-3246671-supplementary.pdf]

Supplementary Materials:

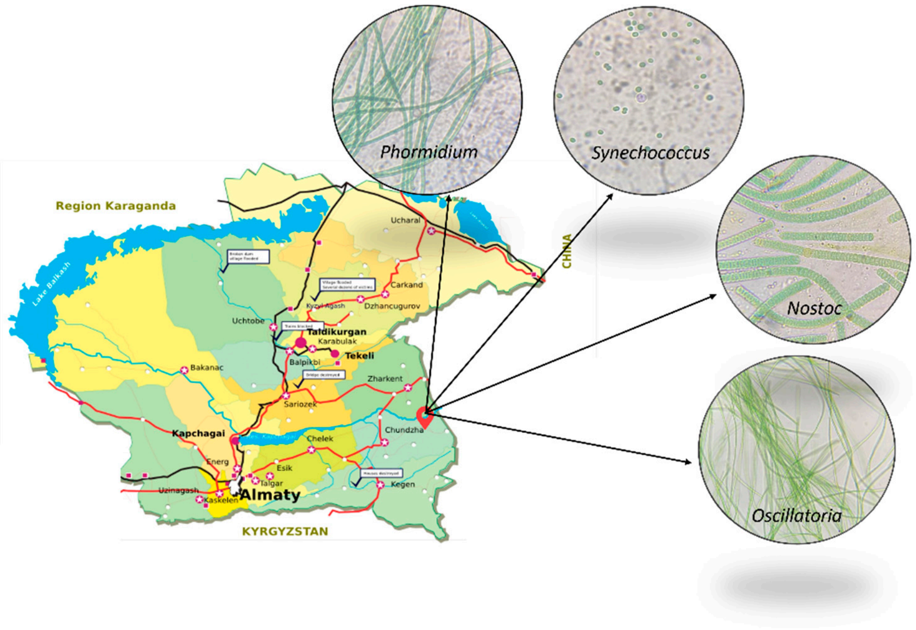

Figure S1. Geographical illustration of sampling points from Chundzha hot springs (GeoCoordinates: 43°32'14.9"N 79°27'56.0"E)

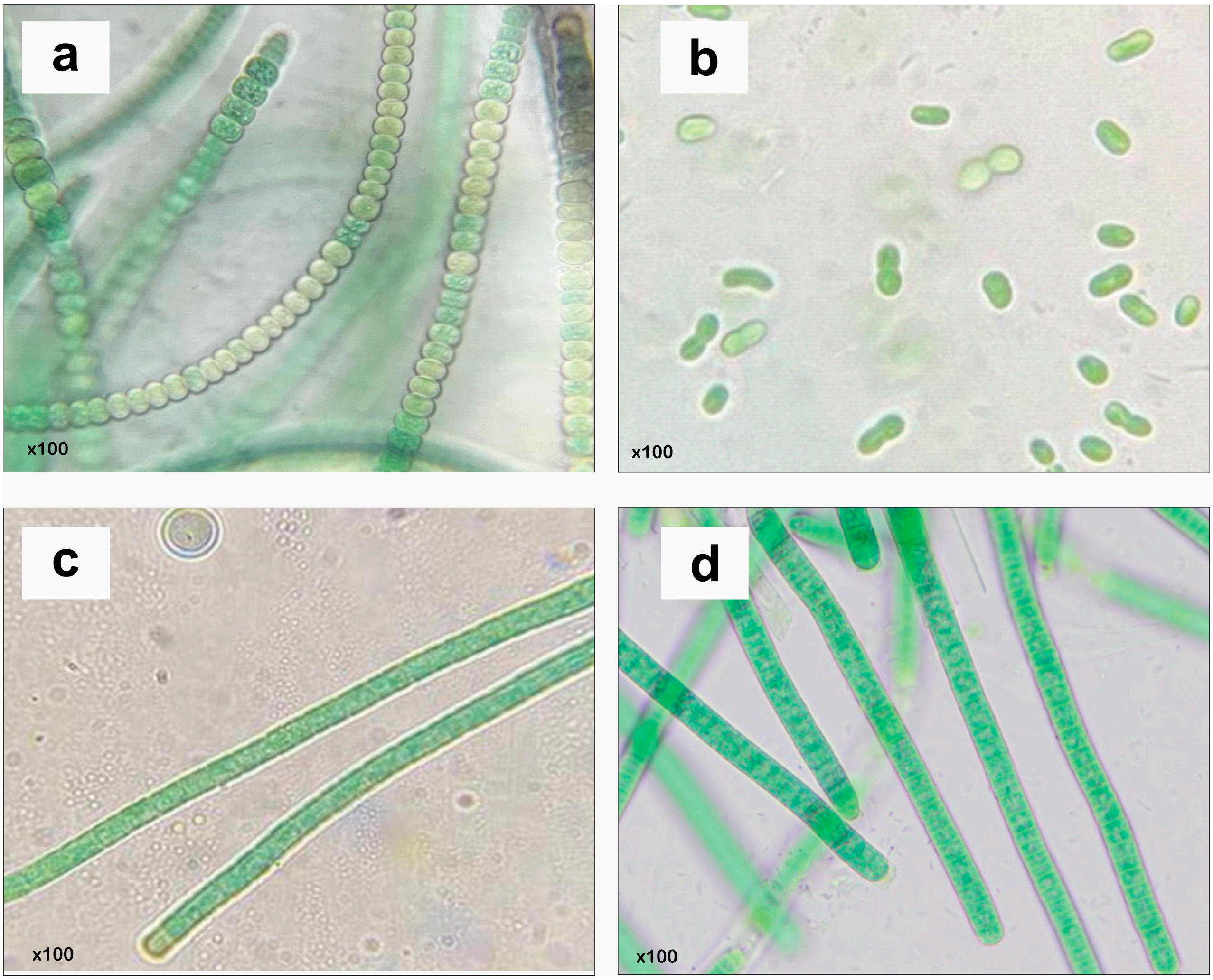

Figure S2. Light microscopy micrographs of (a) *Nostoc calcicola* TSZ 2203, (b) *Synechococcus* sp. CZS 2204, (c) *Phormidium ambiguum* CZS 2205,(d) *Oscillatoria subbrevis* CZS 2201.

Table S1. The taxonomic position and the culture conditions of the maintenance of novel cyanobacterial isolates

Cyanobacterial strain	Family, order	Morphology	Nutrient media	Cultivation conditions
Oscillatoria subbrevis CZS 2201	Oscillatoriaceae, Oscillatoriales	filamentous	Zarrouk	T:32°C, pH:8.5, LI: 62 µE, H: 60%, C_N: 2.5 g L^{-1}
Phormidium ambiguum CZS 2205	Phormidiaceae, Oscilatoriales	filamentous	Zarrouk	T:30°C, pH:8.5, LI: 60 µE, H: 60%, C_N: 2.5 g L^{-1}
Synechococcus sp. CZS 2204	Synechococcaceae, Chroococcales	unicellular	BG-11	T:28°C, pH:7.5, LI: 59 µE, H: 60%, C_N: 1.5 g L^{-1}
Nostoc calcicola TSZ 2203	Nostocaceae, Nostocales	filamentous	$BG\text{-}11_0$	T:28°C, pH:7.5, LI: 53 µE, H: 60%, C_N:0 g L^{-1}
